# Failure to Confirm Typical Echocardiographic Findings of Cardiac Amyloidosis in an Unresolved Nonischemic Cardiomyopathy Associated With Smoldering Multiple Myeloma: A Case Report

**DOI:** 10.7759/cureus.44845

**Published:** 2023-09-07

**Authors:** Carlos Gaibor, Endurance Evbayekha, Brittany Dixon

**Affiliations:** 1 Internal Medicine, St. Luke's Hospital, Chesterfield, USA; 2 Cardiology, St. Luke's Hospital, Chesterfield, USA

**Keywords:** mri cardiac, restrictive cardiomyopathy, smoldering multiple myeloma, amyloidosis, nonischemic cardiomyopathy

## Abstract

Amyloidosis is a leading cause of infiltrative cardiomyopathy and in turn heart failure with preserved ejection fraction. Amyloidosis is mainly classified into amyloid light chain (AL) or primary amyloidosis and transthyretin amyloidosis (ATTR) that is subdivided into wild-type ATTR (ATTRwt) and hereditary or familial transthyretin-related amyloidosis (hATTR). Moreover, strain preservation pattern in the left ventricular apex in echocardiography suggests cardiac amyloidosis and cardiac magnetic resonance (CMR) could identify an infiltrative process. Similarly, the radiotracer uptake of technetium-99m pyrophosphate by myocardium could indicate transthyretin accumulation. In contrast, serum-free light chain (FLC) alongside serum and urine immunoelectrophoresis could indicate AL amyloidosis. Here, we present a case of a 60-year-old male with a classical apical sparing on echocardiography but with an unremarkable CMR and technetium-99 m pyrophosphate.

## Introduction

Heart failure (HF) affects approximately 5.1 million individuals in the United States (US), and the lifetime risk of developing HF is 20% for Americans older than 40 years old. Similarly, its incidence increases with age, rising from approximately 20 per 1000 among those aged 65 to 69 years old to more than 80 per 1000 in those older than 85 years old [1]. HF is a chronic and progressive clinical syndrome characterized by typical signs and symptoms, including shortness of breath, fatigue, elevated jugular venous pressure, rales, and peripheral edema, among others. It is caused by structural or functional cardiac abnormalities resulting in reduced cardiac output and/or elevated intracardiac pressures at rest or during stress [1].

HF is classified according to cardiac ejection fraction (EF). Hence, HF with reduced EF (HFrEF) is defined as EF less than 40%, HF with preserved EF (HFpEF) as EF more than 50%, while HF with borderline EF is defined as EF between 41-49% [2]. Furthermore, HFpEF affects approximately 3 million people in the US and patients are hospitalized approximately 1.4 times per year with an annual mortality rate of approximately 15%. Around 35% of patients with HFpEF present with unexplained dyspnea on exertion and physical, radiographic, and even echocardiographic signs may not be evident at early stages but most patients with HFpEF have a history of hypertension [3]. Cardiac amyloidosis is caused by extracellular deposition of amyloid, which is an insoluble protein, and is one of the leading causes of restrictive cardiomyopathy and, in turn, HFpEF. Amyloidosis is mainly classified into amyloid light chain (AL) or primary amyloidosis and transthyretin amyloidosis (ATTR). AL is caused by the accumulation of monoclonal protein (light chain of immunoglobulins) and is related to plasma cell dyscrasias such as multiple myeloma, Waldenström macroglobulinemia, among others. In contrast, ATTR is caused by the deposition of transthyretin (TTR). TTR, formerly known as pre-albumin, is synthesized in the liver and serves as a transporter of thyroxine and retinol-binding protein [4]. ATTR is further subdivided into wild-type ATTR (ATTRwt) and variant ATTR, caused by point mutations in the TTR gene, generally known as hereditary or familial transthyretin-related amyloidosis (hATTR) [5]. hATTR is autosomal dominant, with the substitution Val30Met being the most frequent mutation [6]. HFpEF can be diagnosed with an echocardiogram, which could indicate cardiac amyloidosis when a "cherry-like" strain preservation pattern in the left ventricular apex (compared with other segments) and a ratio of apical longitudinal strain versus basal and mid-longitudinal strain over 1.3 are found [7-8]. Similarly, prior studies have shown that apical sparing has a sensitivity of 93% and specificity of 82% for cardiac amyloidosis [9]. Cardiac magnetic resonance (CMR) could identify an infiltrative process, whereas technetium-99m pyrophosphate is a radiotracer that suggests TTR using single-photon emission computed tomography (SPECT) [10-11]. In addition, AL amyloidosis is diagnosed with serum-free light chain (FLC) measurements alongside serum and urine immunoelectrophoresis [12].

## Case presentation

The patient is a 60-year-old male who presented with shortness of breath that started 3 hours ago. Despite being a long-time smoker, he denied any significant past medical history. The patient mentioned having been intermittently breathless over the last month but he did not seek medical attention. However, this episode was worse and prompted him to come to the emergency department (ED). During initial evaluation in the ED, the patient was found to be tachycardic with a heart rate of 129 beats per minute, hypertensive with a blood pressure of 216/119 mmHg, and slightly tachypneic with a respiratory rate of 20. Consequently, he experienced a sudden episode of significant hemoptysis, characterized by the presence of frothy sputum and his oxygen saturation dropped to 70%; as a result, intubation was performed alongside with sedation. Moreover, a nitroglycerin drip was ordered and the patient was transferred to the intensive care unit for further management. Initial laboratory revealed a leukocytosis of 17.9 K/uL (normal range 4.3-10.0 K/uL), procalcitonin of 0.44 mg/mL (normal range 0.10-0.24 mg/mL, indicating discouragement of antibiotics; 0.25-0.49 mg/mL, indicating encouragement of antibiotics), which increased to 1.44 mg/mL the following day, and a lactic acid of 2.8 mmol/L (normal range 0.7-2.1 mmol/L) (Table 1).

**Table 1:** Laboratory HDL: high-density lipoprotein; LDL: low-density lipoprotein

Laboratory	Result	Normal range
White blood cells	17.9 K/uL	4.3-10.0 K/uL
Hemoglobin	15.3 g/dl	13.6-16.5 g/dl
Hematocrit	46.00%	40.0-48.0%
Mean corpuscular volume (MCV)	90.2 fL	82.0-99.0 fL
Mean corpuscular hemoglobin (MCH)	30.0 pg	27.2-32.6 pg
Mean corpuscular hemoglobin concentration (MCHC)	33.3 g/dl	31.5-35.5 g/dl
Red cell distribution width (RDW)	15.40%	11.5-14.5%
Sodium	138 mmol/L	137-145 mmol/L
Potassium	4.8 mmol/L	3.5-4.9 mmol/L
Chloride	109 mmol/L	98-107 mmol/L
Magnesium	2.1 mg/dl	1.6-2.6 mg/dl
Bicarbonate	20 mmol/L	22-30 mmol/L
Blood urea nitrogen (BUN)	19 mg/dl	9-20 mg/dl
Creatinine	1.4 mg/dl	0.7-1.3 mg/dl
Glucose	109 mg/dl	74-106 mg/dl
Calcium	9.8 mg/dl	8.4-10.2 mg/dl
Protein total	6.2 g/dl	6.5-8.6 g/dl
Albumin	4.5 g/dl	3.5-5.0 g/dl
Alkaline phosphatase	137 U/L	38-126 U/L
Bilirubin, total	1.1 mg/dl	0.2-1.3 mg/dl
Aspartate aminotransferase (AST)	63 U/L	14-54 U/L
Alanine transaminase (ALT)	31 U/L	≤50 U/L
Lactic acid	2.8 mmol/L	0.7-2.1 mmol/L
Hemoglobin A1C	5.30%	4.0-6.0%
pro-BNP	7850 pg/ml	< 400 pg/ml
Total cholesterol	116 mg/dl	<199 mg/dl
Triglyceride	67 mg/dl	<= 149 mg/dl
Direct HDL	53 mg/dl	>= 41 mg/dl
LDL	50 mg/dl	<=99 mg/dl
Hepatitis B surface antigen	negative	
Hepatitis B core antibody	negative	
Hepatitis C antibody	negative	
HIV-1/2 antigen/antibody	negative	

**Figure 1:**
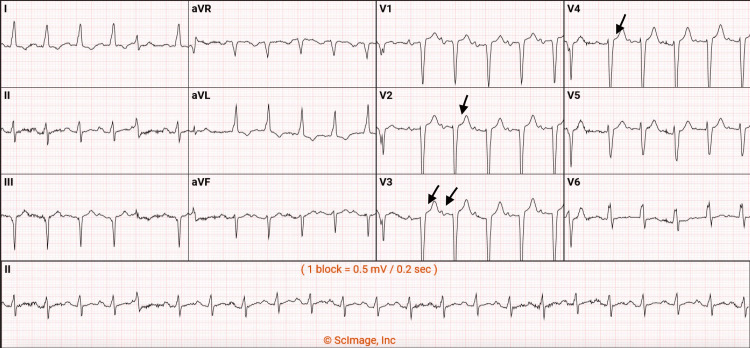
Electrocardiogram showing unspecific ST segment and T wave changes in V2 to V4.

In addition, troponin was found to be slightly elevated at 0.17 ng/mL (normal range is less than 0.12 ng/mL). It increased to 0.22 ng/mL and later to 0.32 ng/mL. These increases were associated with unspecific ST segment and T wave changes in the electrocardiogram. The elevated troponin levels were attributed to demand ischemia due to the absence of specific ischemic changes in the electrocardiogram (Figure 1).

**Figure 2:**
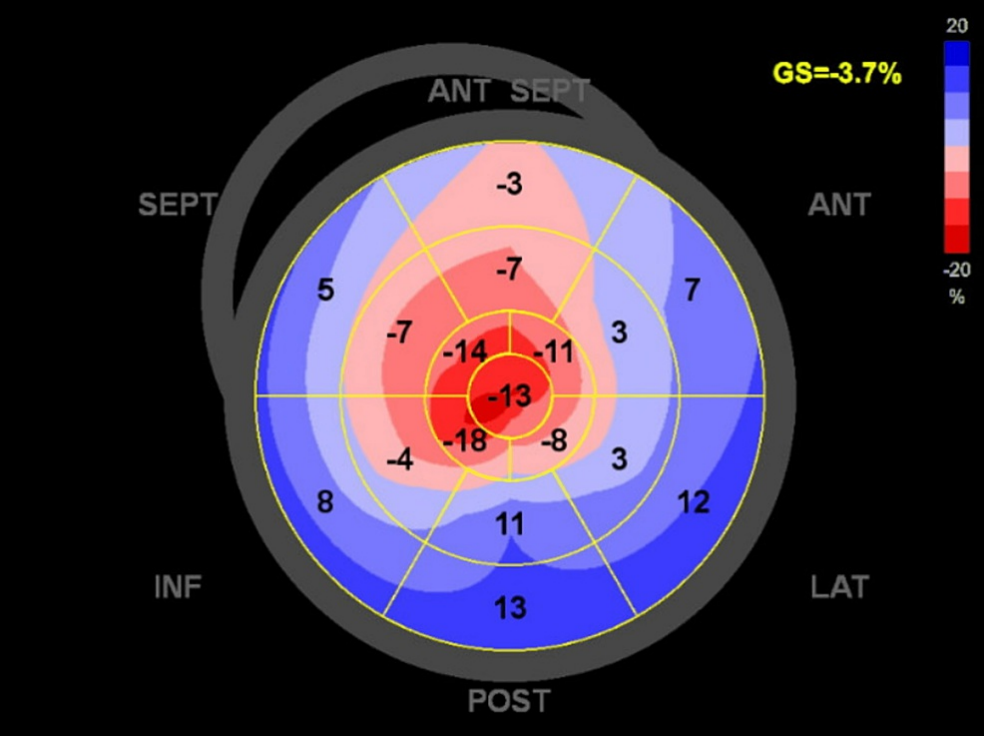
Longitudinal strain bull's eye plot pattern in echocardiogram showing apical sparing

As a result, the blood pressure improved, leading to the discontinuation of nitroglycerin. Instead, an intravenous drip of furosemide and intravenous metoprolol were prescribed. While bronchoscopy did not uncover any signs of intrapulmonary hemorrhage, the echocardiogram displayed a relative preservation of left ventricular strain at the apex with markedly abnormal left ventricular strain in the basal and mid-cavity segments, presenting in a bull's-eye pattern associated with an ejection fraction of 60%, a characteristic finding in restricted cardiomyopathy specifically in cardiac amyloidosis (Figure 2).

**Figure 3:**
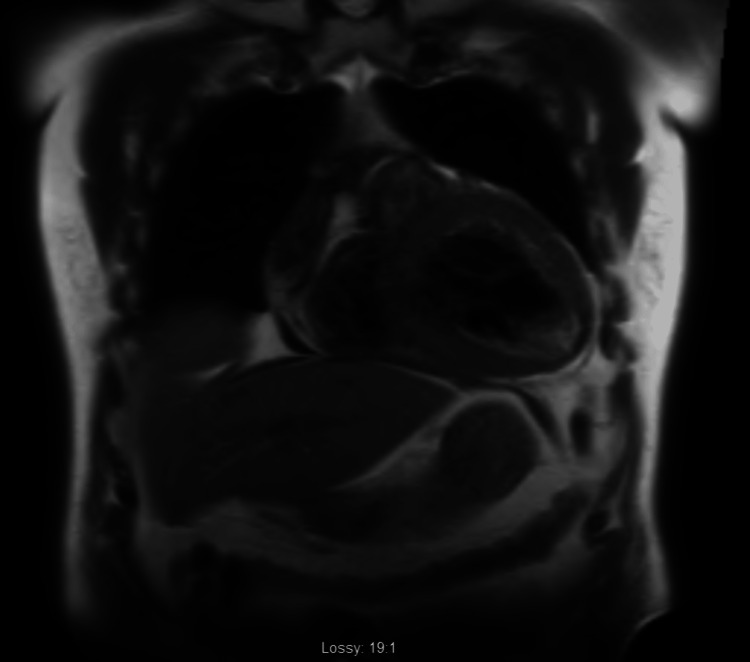
Coronal view of CMR showing no late gadolinium enhancement CMR: Cardiac magnetic resonance

**Figure 4:**
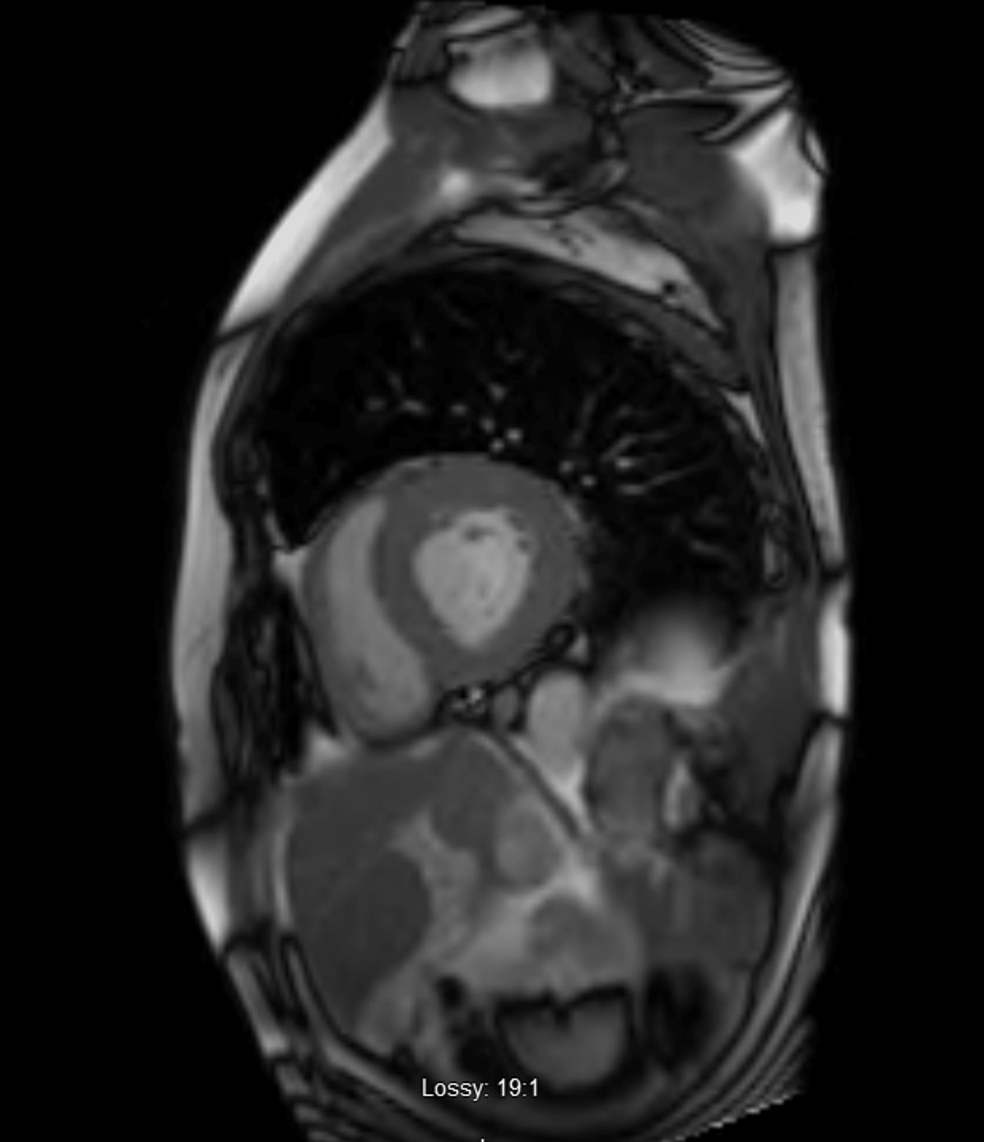
Sagittal view of CMR showing no late gadolinium enhancement CMR: Cardiac magnetic resonance

**Figure 5:**
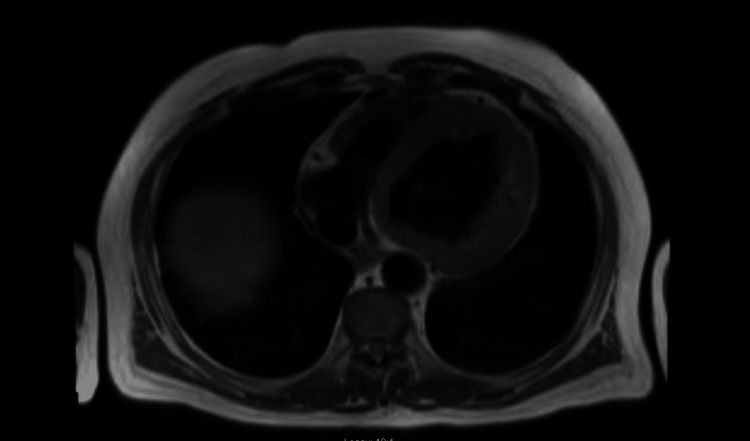
Transversal view of CMR showing no late gadolinium enhancement CMR: Cardiac magnetic resonance

**Figure 6:**
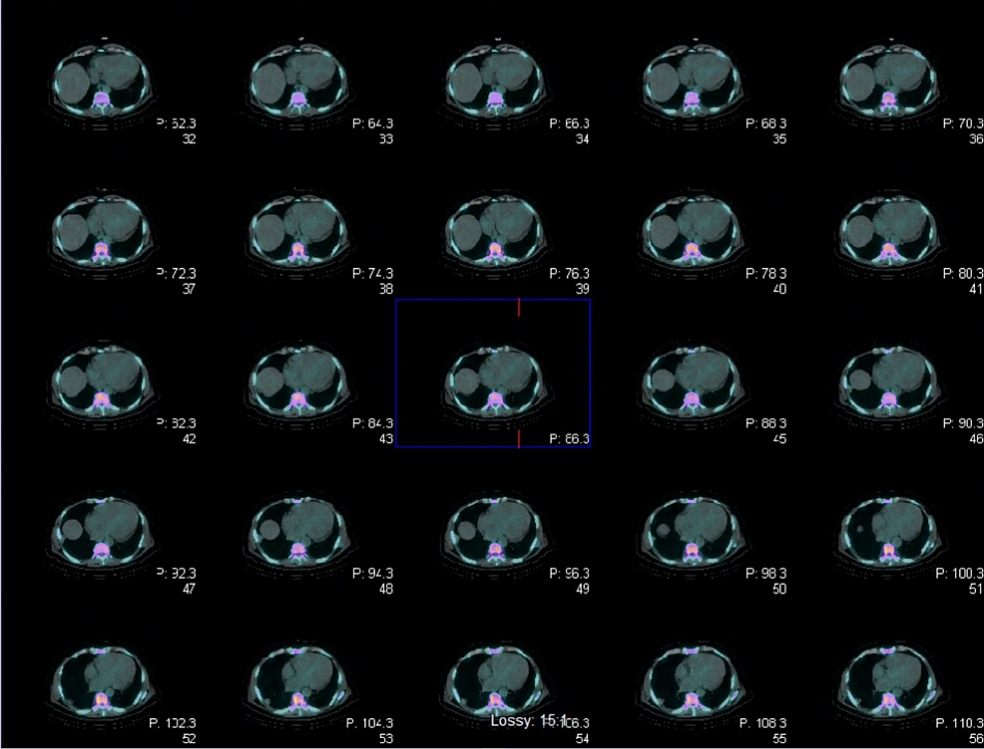
Technetium-99m Pyrophosphate Imaging showing no myocardial tracer uptake

During his stay in the intensive care unit (ICU), the patient developed various episodes of non-sustained tachycardia, so amiodarone was added to his regimen and received a five-day antibiotic course with piperacillin/ tazobactam and vancomycin. On day 4 of hospitalization, the patient was successfully extubated and the following day he was transferred to telemetry where GDMT (Guideline Directed Medical Therapy) was initiated. Furthermore, cardiac catheterization did not reveal any severe obstructive coronary artery (CA) disease. Therefore, nonischemic cardiomyopathy work-up was ordered including cardiac magnetic resonance imaging (MRI) for ruling out CA (Figures 3-5) and technetium pyrophosphate scan for ruling out ATTR amyloidosis (Figure 6) which both were negative.

Despite the serum-free light chains showing an elevated Kappa light chain of 57.5 mg/L (normal range: 3.3-19.4 mg/L) along with an elevated free Kappa/Lambda ratio of 3.21 (normal range: 0.26-1.65), the patient received a diagnosis of smoldering multiple myeloma. This was due to the bone marrow biopsy results, which indicated less than 20% of plasmacytoid cells, an M protein level of less than 2, and a light chain ratio of less than 20. Consequently, the patient was discharged and is currently receiving guideline-directed medical therapy (GDMT) (Tables 2-4).

**Table 2:** Serum Protein Electrophoresis

Serum Protein Electrophoresis	Value	Normal Range
Albumin Fraction	4.56 g/dl	3.77-5.94 g/dl
Alpha 1 globulin	0.14 g/dl	0.06-0.26 g/dl
Alpha 2 globulin	0.95 g/dl	0.47-1.03 g/dl
Beta globulin	0.7 g/dl	0.47-1.06 g/dl
Gamma Globulin	1.55 g/dl	0.50-1.30 g/dl
Aminomethyltransferase monoclonal protein	1.2 g/dl	< 2 gr/dl

**Table 3:** Kappa/Lambda Light Chains Free with Ratio and Immunoglobulins

	Value	Normal Range
Kappa Light Chain, Free	77.1 mg/L	3.3-9.4 mg/L
Lambda Light Chain, Free	17.4 mg/L	5.7-26.3 mg/L
Kappa/Lambda Ratio, Free	4.43	0.26-1.65
Ig G (immunoglobulin G)	1998 mg/dl	603-1613 mg/dl
Ig A (Immunoglobulin A)	203 mg/dl	90-386 mg/dl
IgM (Immunoglobulin M)	50 mg/dl	20-172 mg/dl

**Table 4:** Urine Protein

	Value	Normal range
Urine protein 24 hours	98 mg/24 hours	42-225 mg/24 hours
Protein urine random	12 mg/dl	0-12 mg/dl

## Discussion

This patient, who had no significant past medical history except for being a longtime smoker, presented with severe shortness of breath. Initially, the patient exhibited characteristic signs of flash pulmonary edema, including severe hypertension, tachycardia, and tachypnea. This was accompanied by a large volume of hemoptysis, leading to a drop in oxygen saturation. As a result, the patient had to be intubated, and nitroglycerin infusion was initiated, resulting in significant improvement in hemodynamic parameters. While bronchoscopy did not reveal any signs of intra-alveolar hemorrhage, echocardiography indicated typical findings consistent with cardiac amyloidosis. These findings included apical sparing in conjunction with a preserved ejection fraction. Thus, the patient received a diagnosis of heart failure with preserved ejection fraction. Ischemic causes were ruled out due to unrevealing findings on cardiac catheterization, so non-ischemic etiologies were sought. Although cardiac magnetic resonance imaging failed to detect any infiltrative process, and the technetium pyrophosphate scan did not reveal any myocardial radiotracer uptake, ruling out transthyretin-related amyloidosis, the patient was ultimately diagnosed with smoldering multiple myeloma, known as a risk factor for amyloidosis AL. Therefore, this unique case showed that the characteristic echocardiographic findings for cardiac amyloidosis could be caused by another condition, as this finding is not 100% accurate, as found in previous studies [9]. Even though current sophisticated imaging tests have assisted in the identification of cardiac amyloidosis without the need for cardiac biopsy, some cases like this one still warrant tissue biopsy to elicit the final diagnosis. This case highlights the importance of being cognizant of the sensitivity and specificity of different kinds of cardiac imaging tests. This patient, who was diagnosed with non-ischemic cardiomyopathy and smoldering multiple myeloma, needs to be considered for a cardiac or tissue biopsy for a final diagnosis.

## Conclusions

Non-ischemic cardiomyopathy is always a diagnostic challenge. Here, we present a unique case where the characteristic echocardiography changes for cardiac amyloidosis failed to correlate with confirmatory test. Although the patient was ultimately diagnosed with smoldering multiple myeloma, cardiac magnetic resonance imaging and technetium pyrophosphate scan did not confirm an infiltrative process or transthyretin-related amyloidosis respectively. As a result, this patient with a non-ischemic heart failure with preserved ejection fraction should be considered for a tissue biopsy and genetic testing to elucidate his diagnosis.

## References

[1] Yancy CW, Jessup M, Bozkurt B (2013). 2013 ACCF/AHA guideline for the management of heart failure: executive summary: a report of the American College of Cardiology Foundation/American Heart Association Task Force on practice guidelines. Circulation.

[2] Ponikowski P, Voors AA, Anker SD (2016). 2016 ESC Guidelines for the diagnosis and treatment of acute and chronic heart failure: The Task Force for the diagnosis and treatment of acute and chronic heart failure of the European Society of Cardiology (ESC) developed with the special contribution of the Heart Failure Association (HFA) of the ESC. Eur Heart J.

[3] Redfield MM, Borlaug BA (2023). Heart failure with preserved ejection fraction: a review. JAMA.

[4] Siddiqi OK, Ruberg FL (2018). Cardiac amyloidosis: an update on pathophysiology, diagnosis, and treatment. Trends Cardiovasc Med.

[5] Tian Z, Ren C, Huo L (2020). Wild type transthyretin amyloidosis, a reason not to be forgotten for heart failure of preserved ejection fraction in the elderly. J Geriatr Cardiol.

[6] Finsterer J, Iglseder S, Wanschitz J, Topakian R, Löscher WN, Grisold W (2019). Hereditary transthyretin-related amyloidosis. Acta Neurol Scand.

[7] Pagourelias ED, Mirea O, Duchenne J (2017). Echo parameters for differential diagnosis in cardiac amyloidosis: a head-to-head comparison of deformation and nondeformation parameters. Circ Cardiovasc Imaging.

[8] Senapati A, Sperry BW, Grodin JL (2016). Prognostic implication of relative regional strain ratio in cardiac amyloidosis. Heart.

[9] Kyrouac D, Schiffer W, Lennep B (2022). Echocardiographic and clinical predictors of cardiac amyloidosis: limitations of apical sparing. ESC Heart Fail.

[10] Masri A, Bukhari S, Ahmad S (2020). Efficient 1-hour Technetium-99m pyrophosphate imaging protocol for the diagnosis of transthyretin cardiac amyloidosis. Circ Cardiovasc Imaging.

[11] Poterucha TJ, Elias P, Bokhari S (2021). Diagnosing transthyretin cardiac amyloidosis by Technetium Tc 99m pyrophosphate: a test in evolution. JACC Cardiovasc Imaging.

[12] Velayutham R, Parale C, Sukumaran SK, Anantharaj A (2022). Cardiac amyloid as a presenting feature of multiple myeloma. QJM.

